# CD94-driven *in vitro* expansion of highly functional adaptive NKG2C^+^ NKG2A^-^ CD57^+^ NK cells from CMV^+^ healthy donors

**DOI:** 10.3389/fimmu.2025.1481745

**Published:** 2025-01-31

**Authors:** Chiara Giordano, Simona Carlomagno, Michela Falco, Claudia Cantoni, Massimo Vitale, Ignazio Caruana, Johannes Dirks, Alberto Serio, Letizia Muccio, Giulia Bartalucci, Alessandra Bo, Franco Locatelli, Cristina Bottino, Simona Sivori, Mariella Della Chiesa

**Affiliations:** ^1^ Department of Experimental Medicine, University of Genoa, Genoa, Italy; ^2^ Department of Medicine (DMED), University of Udine, Udine, Italy; ^3^ Department of Services, IRCCS Istituto Giannina Gaslini, Genova, Italy; ^4^ U.O. Patologia e Immunologia sperimentale, IRCCS Ospedale Policlinico San Martino, Genoa, Italy; ^5^ Hematology, Oncology and Stem Cell Transplantation Unit, Department of Pediatrics, University Hospital Würzburg, Würzburg, Germany; ^6^ Department of Pediatrics, University Hospital Würzburg, Würzburg, Germany; ^7^ Hematology and Cell Therapy, IRCCS Ospedale Policlinico San Martino, Genoa, Italy; ^8^ Unit of Hematology/Oncology, Cell and Gene Therapy, Bambino Gesù Children’s Hospital, IRCCS, Rome, Italy; ^9^ Department of Life Sciences and Public Health, Catholic University of the Sacred Heart, Rome, Italy; ^10^ Direzione Scientifica, IRCCS Ospedale Policlinico San Martino, Genoa, Italy

**Keywords:** adaptive NK cells, CD94/NKG2C, expansion, monoclonal antibody, immunotherapy, ADCC, KIR, CMV

## Abstract

**Background:**

Adaptive human natural killer (NK) cells are an NK cell subpopulation arising upon cytomegalovirus (CMV) infection. They are characterized by CD94/NKG2C expression, a mature CD57^+^KIR^+^NKG2A^–^ phenotype, a prolonged lifespan, and remarkable antitumor functions. In light of these features, adaptive NK cells represent suitable candidate to design next-generation therapies, based on their enhanced effector function which could be further boosted by Chimeric Antigen Receptors-engineering, or the combination with cell engagers. For therapeutic approaches, however, it is key to generate large numbers of functional cells.

**Purpose:**

We developed a method to efficiently expand adaptive NK cells from NK-enriched cell preparations derived from the peripheral blood of selected CMV-seropositive healthy donors. The method is based on the use of an anti-CD94 monoclonal antibody (mAb) combined with IL-2 or IL-15.

**Results:**

By setting this method we were able to expand high numbers of NK cells showing the typical adaptive phenotype, CD94/NKG2C^+^ CD94/NKG2A^-^ CD57^+^, and expressing a single self-inhibitory KIR. Expanded cells maintained the CMV-induced molecular signature, exhibited high ADCC capabilities and degranulation against a HLA-E^+^ target. Importantly, mAb-expanded adaptive NK cells did not upregulate PD-1 or other regulatory immune checkpoints that could dampen their function.

**Conclusions:**

By this study we provide hints to improve previous expansion methods, by eliminating the use of genetically modified cells as stimulators, and obtaining effectors not expressing unwanted inhibitory receptors. This new protocol for expanding functional adaptive NK cells is safe, cost-effective and easily implementable in a GMP context, suitable for innovative immunotherapeutic purposes.

## 1 Introduction

Natural Killer (NK) cells are cytotoxic lymphocytes that belong to the innate lymphoid cell (ILC) compartment, capable of rapidly killing tumor or virus-infected cells and regulating immune responses through cytokine and chemokine release (1, 2). These abilities have placed NK cells at the core of novel cell-based immunotherapeutic strategies, especially in the allogeneic setting for refractory oncological patients (3–6).

NK cell function is regulated by the differential triggering of activating and inhibitory receptors (7). The latter include receptors that recognize HLA class I (HLA-I) molecules, i.e., the CD94/NKG2A heterodimer and the inhibitory Killer-cell Immunoglobulin-like Receptors (iKIRs) (8, 9). In particular, the CD94/NKG2A heterodimer is conserved and recognizes the non-classical HLA-E molecule, while iKIRs are highly polymorphic and bind epitopes of classical HLA-I molecules (HLA-A, -B and –C). Specifically, KIR2DL1 recognizes HLA-C allotypes carrying Lysine at position 80 (C2 epitope), KIR2DL2/L3 recognizes HLA-C molecules sharing Asparagine at the same position (C1 epitope), and KIR3DL1 recognizes HLA-A and HLA-B alleles characterized by the Bw4 epitope (10). Furthermore, NK cells can express activating forms of HLA-I-specific receptors including the CD94/NKG2C heterodimer (CD94/NKG2A counterpart) and activating KIRs that can recognize the same ligands as the inhibitory receptors, with different affinity, and transduce signals through the ITAM-bearing adaptor DAP-12 (11–13). The recognition of self HLA-I molecules by CD94/NKG2A and inhibitory KIRs is fundamental to generate NK cells functionally competent toward tumors and infected cells but tolerant to self, through a process called “education” or “licensing” (14). NK cells also express non-HLA-I specific inhibitory receptors that can switch off NK cell function by acting as immune checkpoints (ICs), similarly to iKIR and NKG2A. These additional ICs include PD-1, TIM-3, TIGIT, and LAG3, and can be upregulated or *de novo* expressed by NK cells in pathologic conditions, thus dampening their cytotoxicity (2, 15–17).

In peripheral blood (PB), human NK cells display variegated phenotypes, characterized by different receptor repertoire and function dictated by both genetic factors and environmental stimuli (1, 18). In this context, a common viral infection caused by human cytomegalovirus (CMV) can promote the generation of a peculiar NK cell population, called “memory-like” or adaptive, in both healthy and pathological conditions (19–23). Adaptive NK cells are characterized by the expression of the activating receptor CD94/NKG2C, strong antiviral and antitumor effector functions (especially after antibody-mediated activation) and long-term persistence, an unusual characteristic for cells belonging to innate immunity (24). NKG2C^+^ adaptive NK cells are characterized by a highly differentiated profile, CD56^dim^ CD16^+^ CD57^+^ NKG2A^–^ self iKIR^+^ (i.e. iKIR recognizing self HLA-I molecules), and epigenetic modifications determining an altered expression of transcription factors and adaptor proteins involved in signal transduction. Among these traits, the decreased expression of the adaptor molecule FcεRγ is the most common one in CMV seropositive (CMV^+^) individuals. This feature is associated with greater CD16-dependent cytotoxicity against IgG-opsonized cells, a mechanism known as ADCC (antibody-dependent cellular cytotoxicity) (25–28). Notably, although generated upon a viral infection, adaptive NK lymphocytes can also play an important role against tumors, as suggested by studies that highlighted their protective effects against acute leukemia recurrence (29–32).

In light of their peculiar characteristics, adaptive NKG2C^+^ NK cells hold high potential in cell-based immunotherapies in both infectious diseases and tumors. However, since high numbers of cells are required for immunotherapeutic use, it is key to define expansion methods capable of effectively stimulating the proliferation of functional adaptive NK cells. Such cells could be transferred in patients either alone or in combination with innovative biological tools such as NK cell engagers (NKCE) to maximize their reactivity against tumors or virus-infected cells (4, 6, 33).

The protocols to expand NKG2C^+^ adaptive NK cells previously described (34–37), displayed a good expansion efficiency, but used genetically modified cells and/or induced the expression of inhibitory receptors, disadvantages that can be overcome by the approach proposed here. Our method is based on the use of an epitope specific anti-CD94 monoclonal antibody (mAb) that, in the presence of IL-2 or IL-15, can efficiently induce the expansion of adaptive NKG2C^+^ NK cells, starting from PB-NK cells of CMV^+^ healthy donors. Upon mAb-mediated stimulation, adaptive NK cell expansions display an NKG2A^–^ self KIR^+^ profile and strong ADCC capability, maintaining the distinctive molecular signature of CMV-induced NK cells.

## 2 Methods

### 2.1 PB samples

PB samples from healthy adult donors were provided by the Transfusion Center-IRCCS Ospedale Policlinico San Martino (Genoa, Italy). The donors gave their informed consent to participate in this study, approved by the Ethics Committee of the Liguria Region (Protocol no. 39/2012, number CER Liguria: DB ig 10125), in accordance with the Declaration of Helsinki.

### 2.2 CMV serology and PB sample processing

CMV serology was evaluated on plasma samples obtained from donors PB, using an enzyme immunoassay for virus-specific IgG immunoglobulins (CMV IgG ELISA Kit, Technogenetics, Lodi, Italy).

PB mononuclear cells (PBMCs) were separated by density gradient centrifugation (Ficoll-Hypaque solution, Sigma-Aldrich, St. Louis, MO), analyzed by immunofluorescence and flow-cytometry to evaluate the donor eligibility (CMV serostatus, NK cell frequency in PBMC over 10%) and frozen. Subsequently, PBMC from selected donors (see section 3.1) were thawed and processed as indicated below.

### 2.3 Anti-CD94 mAb

The mAb used in this study recognizes the CD94/NKG2C and CD94/NKG2A heterodimeric complexes on human cells by interacting specifically with the CD94 molecule and was generated and characterized in our laboratory (38–40). The purified mAb used in cell cultures was obtained from supernatants collected after hybridoma culture with serum-free medium (CD Hybridoma, Gibco, ThermoFisher Scientific, Waltham, Massachusetts, USA). MAb purification was performed by affinity chromatography using HiTrap Protein G HP columns (GE Healthcare, Chicago, Illinois, USA).

### 2.4 Cell preparations and cultures

The medium used in cultures and throughout the experiments was RPMI-1640, supplemented with 2mM L-glutamine, 1% penicillin-streptomycin, 10% heat-inactivated Fetal Bovine Serum (FBS, Gibco). Recombinant human (rh) IL-2 (Proleukin, Chiron, Emeryville, CA) or rhIL-15 (Peprotech, London, UK) was added to the medium where indicated (from now on, rh will be omitted).

The protocol developed after testing different experimental conditions, requires the following steps:

1. PBMC isolation from selected CMV^+^ HD characterized by over 20-25% NKG2C^+^ CD56^dim^NK cells (**Supplementary Table S1**);
2. T-cell depletion to obtain NK-enriched cell populations;
3. 14-day culture upon addition on day 0 of a specific anti-CD94 mAb, in the presence of IL-2 or IL-15.

**Figure 1:**
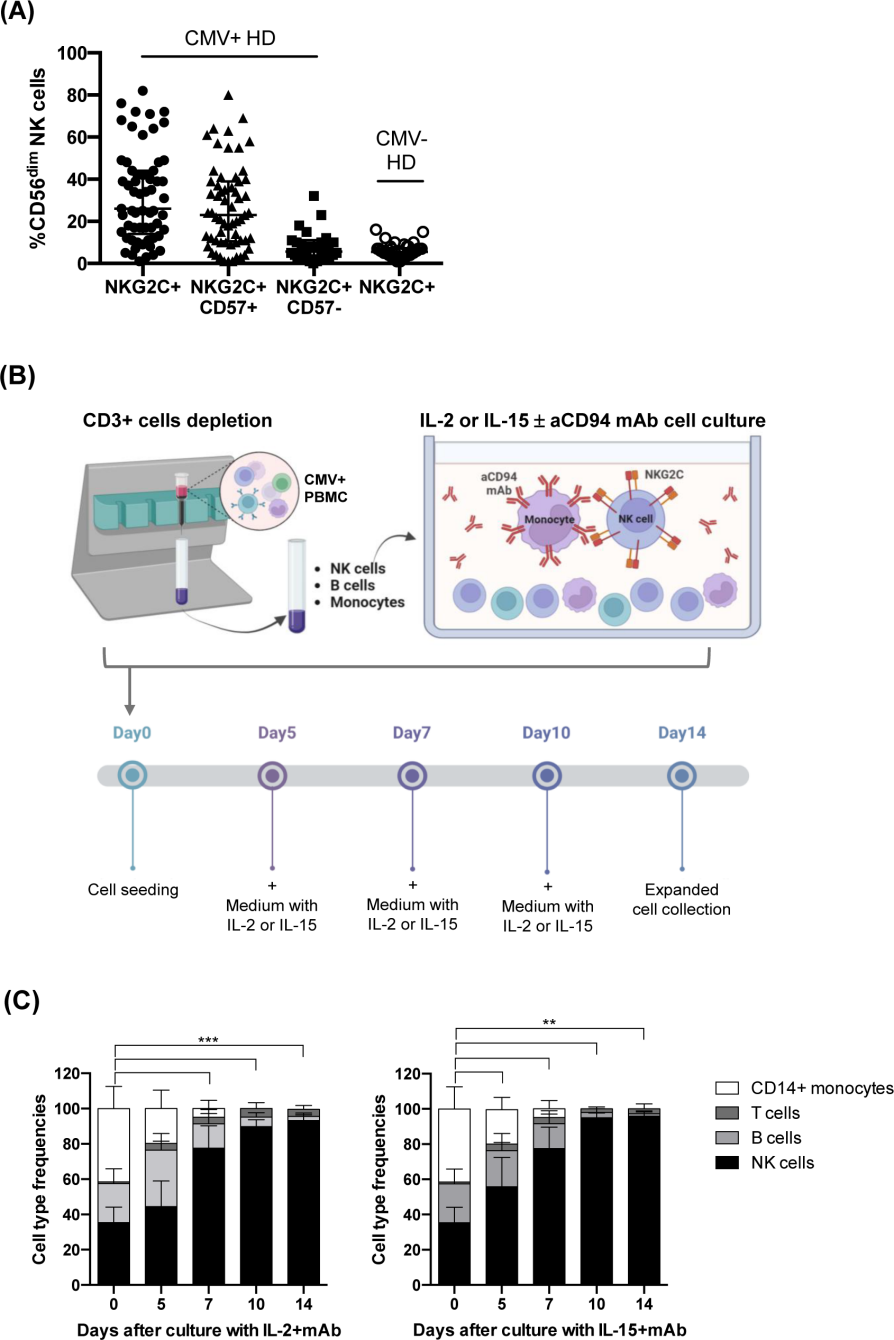
Donor selection and expansion protocol for adaptive NK cells from CMV^+^ healthy subjects. **(A)** Frequency of NKG2C^+^CD56^dim^ NK cells in the PB of healthy CMV^+^ (n=60, solid circles) and CMV^–^ (n=38, empty circles) individuals. For CMV^+^ donors, the frequencies of NKG2C^+^CD57^+^ (triangles) and NKG2C^+^CD57^–^ (squares) NK cell subsets are also shown. Mean frequency ± SD is indicated. **(B)** Adaptive NK cell expansion procedure is depicted. T cell-depleted PBMCs from selected CMV^+^ healthy donors were cultured with anti-CD94 mAb, in the presence of IL-2 or IL-15. This figure was created with BioRender.com. **(C)** The relative frequency of the various cell types in culture (NK, B, T cells, and CD14^+^ monocytes) was evaluated by multiparametric flow cytometry and is reported at the different time points analyzed (day 0, 5, 7 and 14). T-cell depleted PBMCs from selected CMV^+^ donors were cultured as indicated in **(B)** in the presence of anti-CD94 mAb with either IL-2 (left) or IL-15 (right) (n=13 experiments with 10 different donors). The mean frequency ± SD is indicated for each cell population. Differences in NK cell frequencies at day 0 with respect to day 5, 7, 10 and 14 were evaluated using Mann-Whitney test and statistical significances are reported (**p<0.01; ***p<0.001).

In particular, PBMCs obtained from selected donors were depleted of T lymphocytes by positive selection with anti-CD3 magnetic microbeads (MACSmicrobeads Miltenyi Biotec, Bergisch Gladbach, Germany). T cell-depleted PBMCs, enriched in NK cells, were cultured as depicted in **Figure 1B**. Briefly, T cell-depleted PBMCs were cultured in the presence of IL-2 or IL-15 at the final concentrations of 300 IU/ml and 20 ng/ml respectively, in the presence or in the absence of the anti-CD94 mAb, at a final concentration of 0.125 μg/ml, a dosage chosen after testing a range of concentrations from 0.0125 μg/ml to 5 μg/ml. The mAb was added to cultures only at day 0. Cells were seeded at a concentration of 5x10^5^/ml in round 96-well plates (10^5^ post-depletion cells/well). On day 5, fresh medium containing the correct cytokine was added to cultures, while on days 7 and 10 a 40%-50% change of medium was carried out. On days 5, 7, 10 and 14, cells were collected, counted (Trypan Blue, Sigma-Aldrich was used to exclude dead cells) and characterized by flow-cytometry analyses as described in the text.

### 2.5 Proliferation assay

In each experiment a fraction of T cell-depleted PBMCs was labeled with the intracellular fluorescent dye 5 (6)-carboxyfluorescein diacetate succinimidyl ester (CFSE, Molecular Probes, Life Technologies, Thermo Fisher Scientific, California, USA), as previously described (28), and cultured according to the protocol shown in **Figure 1B**. On the indicated days, the cells were subjected to flow cytofluorimetric investigations to evaluate their proliferative abilities indicated by CFSE dilution.

### 2.6 mAbs and flow cytometric analyses

The different mAbs, as well as the isotype-specific secondary reagents used in flow-cytometry analyses, are shown in **Supplementary Table S2** (**Supplementary Table S2**). See **Supplementary File** for details on flow-cytometry procedures and analyses.

### 2.7 Functional assays

On day 14 of culture, the cells expanded in the presence of cytokine+mAb were collected, washed, and incubated with different cell lines at an effector NK cell:target cell ratio of 1:1 for 3 hours, in culture medium supplemented with anti-CD107a mAb-PE as previously described (41). In particular, the following cell lines were used: K562, NALM-16 (B-Cell Precursors Acute Lymphatic Leukemia cell line), HT-29 and LoVo (colorectal cancer cell lines), HL-60 (Acute Myeloid Leukemia cell line), Raji previously opsonized or not with the anti-CD20 mAb Rituximab (1 μg/ml), LCL 721.221 wild type (221wt) and LCL.721.221AEH (221E) (i.e., LCL 721.221 transfected to express HLA-A leader sequence), kind gift from Prof. Miguel Lopez-Botet and Dr. Aura Muntasell (Hospital del Mar Medical Research Institute IMIM and University Pompeu Fabra, Barcelona, Spain), originally developed by Geraghty D. and coworkers (42). Basal degranulation of NK cells was assessed in 3-hour cultures without tumor cell lines. Subsequently, cells were collected, stained and analyzed by flow-cytometry as described in **Supplementary Methods**.

To detect NK intracellular production of IFN-γ and TNF-α, on day 14 mAb-expanded NK cells were washed and incubated with different cell lines. i.e. K562, 221wt, 221E, Raji rituximab-coated or not, or medium alone, an effector NK cell:target cell ratio of 1:1 for 4 hours in the presence of GolgiStop (BD Biosciences Pharmingen). Thereafter, cells were washed, stained as described above for CD107a assays and then fixed and permeabilized with Foxp3 permeabilization buffer Kit (Miltenyi Biotec, Bergisch Gladbach, Germany). IFN-γ and TNF-α production was detected by subsequent intracellular staining with PE-labeled mAbs and cytofluorimetric analysis.

### 2.8 Statistical analysis

Nonparametric Wilcoxon-Mann-Whitney tests were used. Statistical significance (p value) is indicated in the different graphs (*p<0.05; ** p<0.01; *** p<0.001; **** p<0.0001). Median Fluorescence Intensity values were normalized before calculating statistical significance. Graphs and statistical analyses were performed with GraphPad Prism 8 (GraphPad Software, www.graphpad.com).

## 3 Results

### 3.1 CD94-induced proliferation of PB-NK cells from CMV^+^ healthy donors drives adaptive NKG2C^+^ CD57^+^ NK cell expansion

To set up a method efficiently expanding NKG2C^+^ NK cells, we first collected and stored PBMC from several CMV^+^ healthy donors (HD); the selected donors were characterized by a percentage of CD56^dim^NKG2C^+^ NK cells close to the median frequency observed (26%, **Figure 1A**). The gating strategy is shown in **Supplementary Figure 1A** (**Supplementary Figure S1A**). Indeed, the percentage of the NKG2C^+^ NK cell population in PB of CMV^+^ HD ranged from 1 to 82% (**Figure 1A**): most NKG2C^+^ NK cells coexpressed the marker of terminal differentiation CD57, representing the adaptive NKG2C^+^CD57^+^ NK cell subset (median=23%, range =1-80%, **Figure 1A**), while NKG2C^+^ NK cells lacking CD57 were present at lower frequencies in most CMV^+^ HD (median=6%, range=0-32%). In CMV-seronegative (CMV^–^) HD, low amounts of NKG2C^+^ NK cells could be observed (median=6%, range=1-16%, **Figure 1A**) (19, 28).

After testing different experimental conditions, we developed an efficient expansion method summarized in **Figure 1B**. The protocol requires three steps, i.e. PBMC isolation from selected CMV^+^ HD, T-cell depletion to obtain NK-enriched cell populations and 14-day culture in the presence of a specific anti-CD94 mAb, with IL-2 or IL-15.

We selected a mAb produced in our lab that recognizes CD94, the non-transducing monomer that associates with NKG2A or NKG2C to form the inhibitory or activating heterodimeric NK cell receptor, respectively (39, 40). The mAb-mediated engagement of CD94 induces inhibitory signals in CD94/NKG2A^+^ NK cells while triggering activating signals in CD94/NKG2C^+^ NK cells (43, 44). Notably, at variance with previously described methods, our culture protocol did not include stimuli like feeder cells or genetically manipulated HLA-E^+^ transfected cell lines (34, 35, 37). However, a regular addition or substitution of medium with fresh cytokines every 2-3 days was required (**Figure 1B**).

**Figure 2:**
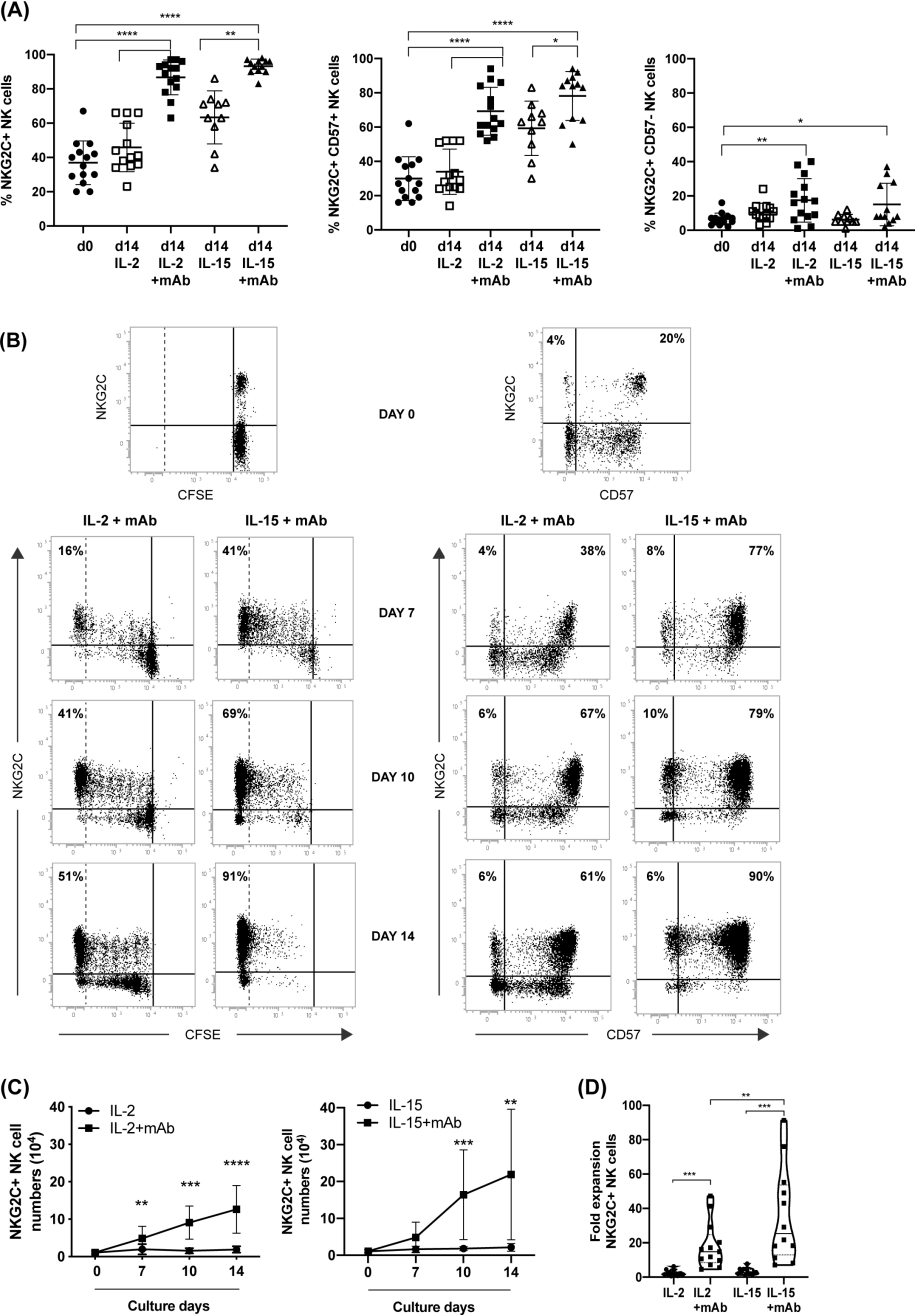
CD94 mAb-induced growth of NKG2C^+^ NK cells by efficient proliferation of NKG2C/CD57 NK cell subsets. **(A)** The frequency of the different NKG2C/CD57 NK cell populations was evaluated by flow-cytometry comparing day 0 (circle) with day 14 of culture with IL-2 (empty square), IL-2+mAb (square), IL-15 (empty triangle) or IL-15+mAb (triangle). The frequency of total NKG2C^+^ (left), NKG2C^+^CD57^+^ (central) or NKG2C^+^CD57^–^ NK cells (right) are shown. Bars indicate the mean ± SD. Differences in NK cell subsets abundance at day 0 with respect to day 14 in IL-2 or IL-15 conditions were evaluated using Mann-Whitney test and statistical significances are reported (*p<0.05; **p<0.01; ***p<0.001; ****p<0.0001). **(B)** T-depleted PBMCs labeled with CFSE were cultured according to the protocol in **Figure 1B** in parallel cultures. The gradual CFSE dilution (left) and the progressive increase of NKG2C^+^CD57^+^ NK cell frequencies (right) are shown at different time points for both IL-2+mAb and IL-15+mAb culture conditions. Percentages of highly proliferating, CFSE negative NKG2C^+^ NK cells (identified by a dotted line) and NKG2C/CD57 NK cell subsets frequencies are reported in the corresponding quadrants. A representative experiment out of 13 is shown. **(C)** The number of NKG2C^+^ NK lymphocytes was evaluated at various culture times starting from day 0, when 10^5^ T-depleted PBMC per well were seeded. Growth curves of cell cultures carried out with (squares) or without (circles) the addition of the anti-CD94 mAb, in the presence of IL-2 (left) or IL-15 (right) are shown. The number of NKG2C^+^ NK lymphocytes was calculated for each experiment (n=13) based on the composition of the cell culture as indicated in **Figure 1C** (gating strategy for cultured NKG2C^+^ NK cells in **Supplementary Figure S1B**). Bars indicate the mean ± SD. Differences in NK cell numbers comparing cultures in the presence and in the absence of the mAb were evaluated for each time point (7, 10, 14 days) using the Mann-Whitney test. Statistical significance is reported at each point (*p<0.05; **p<0.01; ***p<0.001; ****p<0.0001). **(D)** Fold expansion in cell numbers of NKG2C^+^ NK cells in the different culture conditions on day 14 (n=13). Statistical significance is reported (**p<0.01; ***p<0.001).

As shown in **Figure 1C**, upon T-cell depletion and before expansion (day 0), the cell population included NK cells (mean=32%; range 25-52%), B cells (mean=22%; range 10-34%), and monocytes (mean=41%; range 20-60%), while T cells were present at negligible frequencies (mean=0, range 0-1%) (see **Supplementary Figures S1A, B** for gating strategy). Following the mAb-mediated NK stimulation, the frequency of NK cells progressively increased, representing virtually the only cell type found in cultures on days 10 and 14; this occurred in the presence of either IL-2 (**Figure 1C** left) or IL-15 (**Figure 1C** right). In particular, the mAb addition on day 0 significantly increased the frequency of NKG2C^+^ NK cells in both cytokine conditions, driving the selective expansion of NKG2C^+^ NK cells that were predominant on day 14 (**Figure 2A** left). A large fraction of NKG2C^+^ NK cells expanded with cytokine+mAb were represented by adaptive NKG2C^+^ CD57^+^ NK cells (**Figure 2A** middle), while limited numbers of NKG2C^+^ NK cells lacking CD57 were detectable (**Figure 2A** right). In parallel cultures, T-depleted PBMC were labeled with CFSE and stimulated with mAb+either of the two cytokines; the progressive expansion of NKG2C^+^ NK cells, predominantly expressing CD57 (right plots) was also clearly demonstrated, showing a tendency to proliferate more rapidly when IL-15+mAb was used, compared to IL-2+mAb (see representative donor in **Figure 2B**).

Importantly, the NKG2C^+^ NK cell numbers observed upon 10-14 days of culture in the presence of anti-CD94 mAb were significantly higher as compared to cultures carried out without the mAb (**Figure 2C**). We observed a tendency to obtain higher numbers of NKG2C^+^ NK cells in the presence of IL-15+mAb (**Figure 2C** right) compared to IL-2+mAb (**Figure 2C** left) as also suggested by the fold expansion rate reported in **Figure 2D** for the different culture conditions. We did not observe any further cell number increase after day 14 of culture, although NKG2C^+^ NK cells were able to survive longer. Finally, when we cultured T-cell depleted PBMCs from CMV^–^ HD using the same protocol, we did not succeed in expanding high numbers of NKG2C^+^ NK cells (not shown). Interestingly, we observed that the mAb could be eliminated from the culture 5 days after cell seeding, without affecting the expansion of adaptive NK cells (not shown).

### 3.2 Most expanded NKG2C^+^ CD57^+^ NK cells are NKG2A^-^, single self KIR^+^, and do not upregulate PD-1

**Figure 3:**
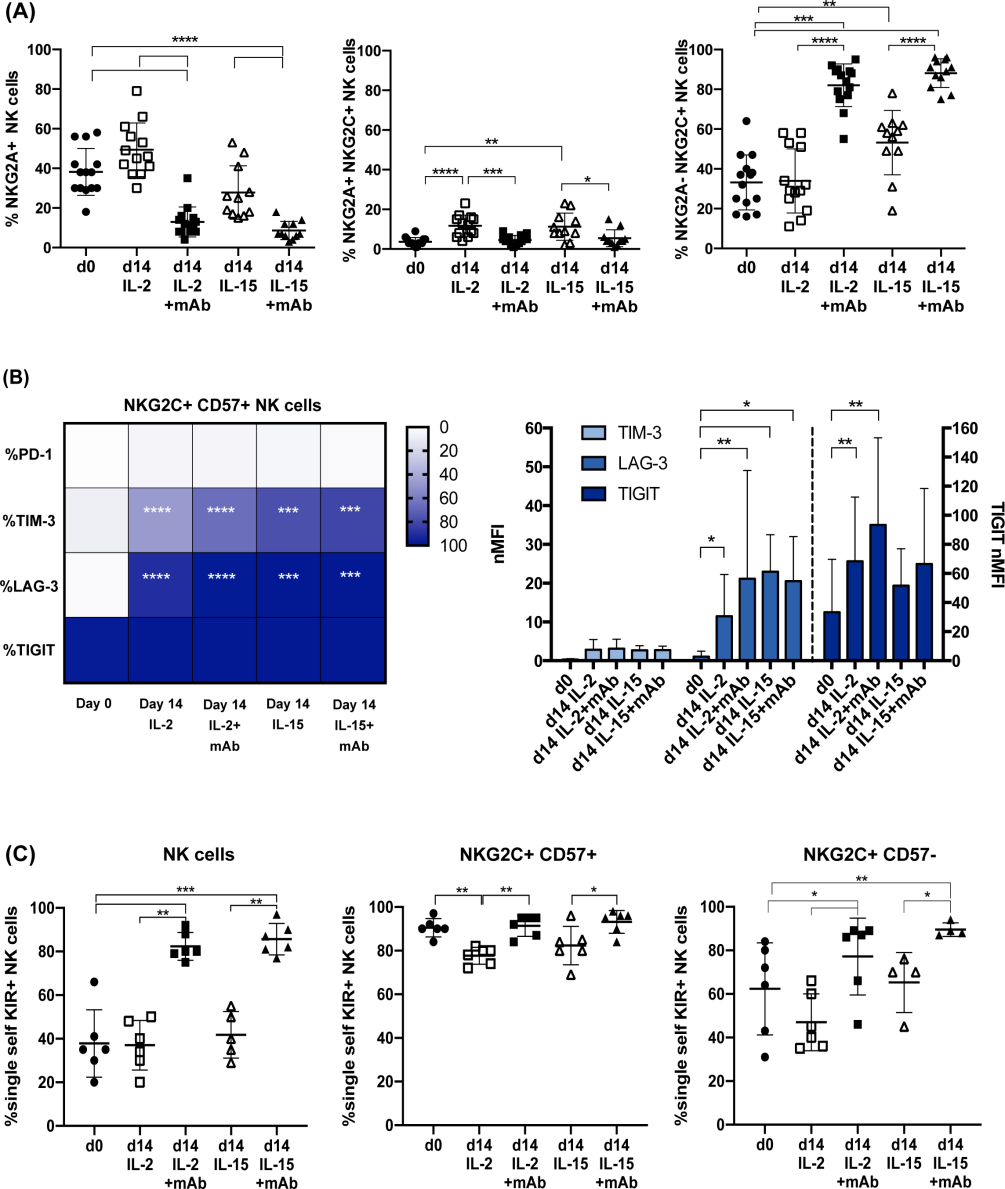
CD94 mAb-induced expansion of NKG2A^–^PD-1^–^ adaptive NKG2C^+^ NK cells expressing a single self iKIR. In **(A)** the frequency of total NKG2A^+^ (left), NKG2A^+^NKG2C^+^ (central) or NKG2A**^–^** NKG2C^+^ NK cells (right), evaluated by flow-cytometry, are shown comparing day 0 (circles) with day 14 of culture with IL-2 (empty squares), IL-2+mAb (squares), IL-15 (empty triangle) or IL-15+mAb (triangles), (n=13). Bars indicate the mean ± SD. Differences in NK cell frequencies were evaluated using Mann-Whitney test and statistical significances are reported (*p<0.05; **p<0.01; ***p<0.001; ****p<0.0001). **(B)** Heatmap representing the percentage of NKG2C^+^CD57^+^ NK cells expressing the indicated immune checkpoint receptors (PD-1, TIM-3, LAG-3, TIGIT) (left panel) and histogram with the normalized Median Fluorescence Intensity (nMFI) of TIM-3, LAG-3, and TIGIT expression by NKG2C^+^CD57^+^ NK cells (right panel) are shown on day 0 and day 14 in both cytokine conditions (IL-2, n=10 donors or IL-15, n= 6 donors), in the presence or absence of anti-CD94 mAb. The different percentages (left) or nMFI (right) at day 0 with respect to day 14, in IL-2 or IL-15, with and without mAb, were compared using the Wilcoxon-Mann-Whitney test (*p<0.05; **p<0.01; ***p<0.001; ****p<0.0001) and statistical significances are reported. No statistically significant differences were observed when the IL-2 vs IL-2+mAb and the IL-15 vs IL-15+mAb conditions were compared. **(C)** The frequency of NK cells expressing exclusively a single self-specific iKIR (KIR2DL1 in n=1, KIR2DL2-L3 in n=5) and lacking NKG2A and the other KIR receptors are shown on all NK cells (left), on NKG2C^+^CD57^+^ (center) and on NKG2C^+^CD57**^–^** (right) NK cell subsets, comparing day 0 with day 14 as in **(A)**, using Mann-Whitney test (gating strategy for single self iKIR^+^ NK cells in **Supplementary Figure S1C**). Bars indicate the mean ± SD and statistical significance is reported (*p<0.05; **p<0.01; ***p<0.001; ****p<0.0001).

The prolonged culture of NK cells in the presence of activating cytokines such as IL-2, IL-12 and IL-15, can induce upregulation of NKG2A and other inhibitory molecules that could decrease the therapeutic potential of NKG2C^+^ NK cells (36, 45). We assessed the NKG2A expression in our culture system (mAb+cytokine) and we did not detect any significant increase of CD94/NKG2A^+^ NK cells, which were even significantly lower if comparing NK cells on day 0 and day 14 (**Figure 3A** left). In addition, we did not observe any expansion/induction of NK cells coexpressing CD94/NKG2C and CD94/NKG2A (**Figure 3A** center). It is thus reasonable to speculate that the CD94 engagement by triggering the NKG2A-mediated inhibitory signal prevented the expansion of both NKG2A^+^ NKG2C^–^ and NKG2A^+^NKG2C^+^ NK cells. Conversely, it efficiently promoted the expansion of NKG2C^+^ cells lacking NKG2A (**Figure 3A** right).

Besides NKG2A, the cytotoxic activity of NK cells can be dampened by other ICs whose expression can be induced or increased following NK cell activation. We focused on the expression of PD-1 and other relevant ICs (i.e., TIM-3, LAG-3 and TIGIT) on expanded NKG2C^+^ NK cells. At variance with other methods, neither the cytokine nor the mAb+cytokine stimulation induced PD-1 expression in NKG2C^+^CD57^+^ NK cells (**Figure 3B**). On the other hand, both TIM-3 and LAG-3, virtually absent on day 0, were significantly upregulated in a relevant fraction of NK cells by exposure to IL-2 or IL-15. However, neither the expression of these ICs nor the frequency of TIM-3^+^ and LAG-3^+^ cells increased significantly upon the addition of the mAb (**Figure 3B**). Finally, TIGIT expression could be detected already at day 0 on most adaptive NK cells and its expression level slightly increased at day 14 after expansion independently from mAb addition, although reaching statistical significance only with IL-2 (**Figure 3B** right).

As mentioned previously, CMV-driven adaptive NK cells are characterized by the expression of self iKIRs, i.e., iKIRs that recognize HLA-I molecules expressed by the individual’s healthy cells. In a subgroup of donors, the *KIR* and *KIR-ligand* genotypes were analyzed and the expansion of single self iKIR^+^NKG2A^–^NKG2C^+^ NK cells upon cytokine+mAb stimulation was assessed. The gating strategy is shown in **Supplementary Figure S1C**. As shown in **Figure 3C** (left), the frequency of NKG2A^–^ NK cells expressing a single self iKIR significantly increased (KIR2DL1 in 1 donor, HD A; KIR2DL3 in 3 donors, HD B, C, and F; KIR2DL2/L3 in 2 donors, HD D and E, **Supplementary Figure S2**). On day 0, virtually all NKG2C^+^CD57^+^ NK cells were NKG2A^–^ single self iKIR^+^, a profile that was maintained upon mAb-mediated expansion (**Figure 3C** center). The frequency of NKG2A^–^ single self iKIR^+^ NK cells significantly increased on day 14 also in the NKG2C^+^ CD57^–^ NK cell subset, whose profile was more heterogeneous on day 0 (**Figure 3C** right). In line with previous studies, we could not find single self KIR3DL1^+^ NKG2C^+^ NK cells either before or after expansion (not shown) (22, 25, 26). Overall, these observations show that our mAb+cytokine protocol selectively expands NKG2C^+^ NKG2A^–^ single self iKIR^+^ NK cells without inducing *de novo* PD-1 expression or further upregulating cytokine-induced TIM-3 and LAG-3 expression.

### 3.3 Expanded adaptive NK cells maintain adaptive traits, display high ADCC abilities, and are active against both HLA-E^+^ targets and different tumor cell lines

**Figure 4:**
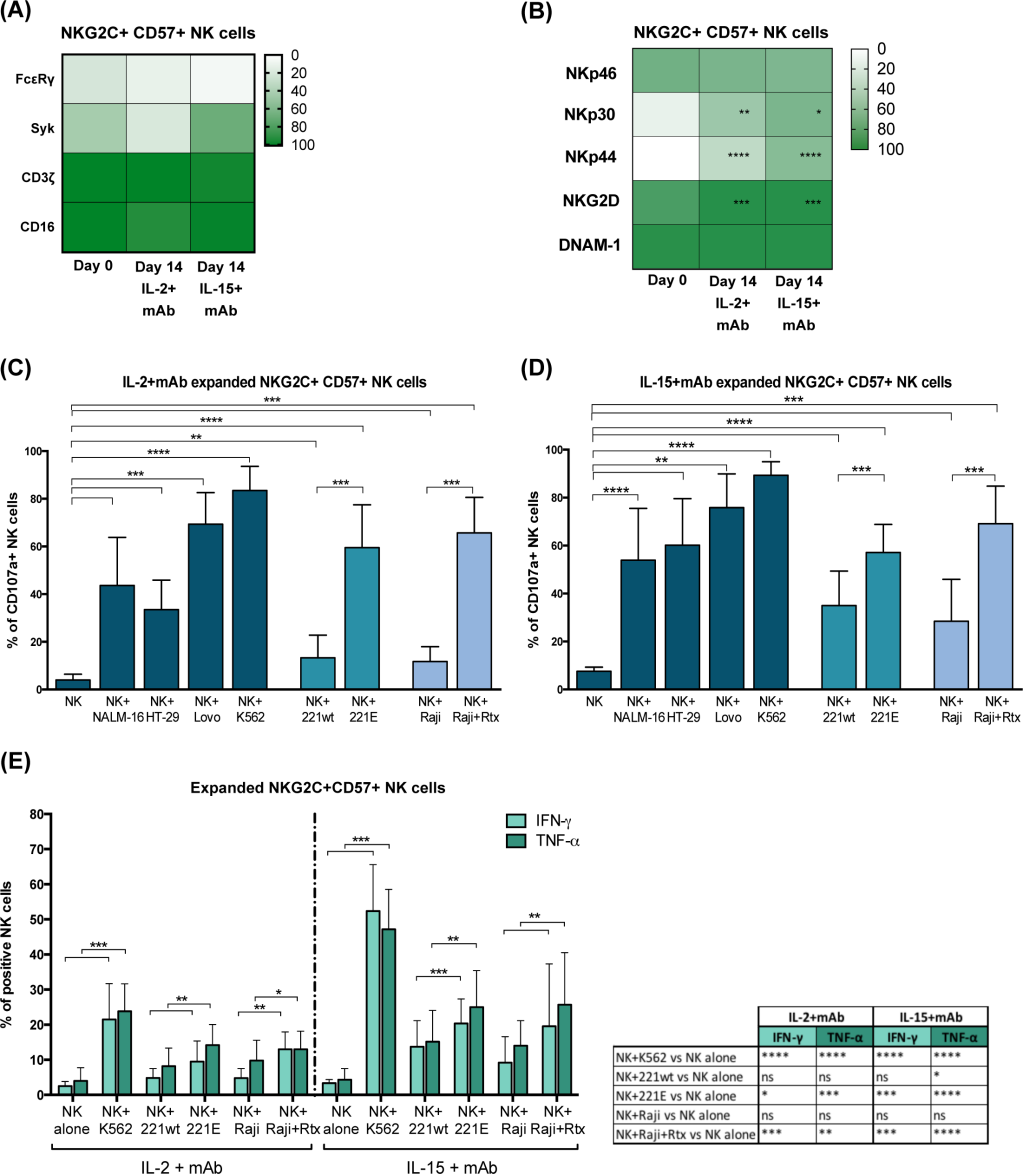
MAb-expanded NKG2C^+^CD57^+^ adaptive NK cells maintain CMV-driven molecular features and high functional abilities. **(A)** Heatmap showing the percentage of NKG2C^+^CD57^+^ NK cells expressing CD16 and the indicated intracellular signaling molecules (FcεRγ, Syk, CD3ζ) at day 0 and 14 in IL2+mAb or IL-15+mAb. No statistically significant differences were observed when day 0 was compared to the IL-2+mAb or IL-15+mAb conditions. **(B)** Heatmap showing the percentage of NKG2C^+^CD57^+^ NK cells expressing the indicated activating receptors (NKp46, NKp30, NKp44, NKG2D, DNAM-1) at day 0 and 14 in IL2+mAb or IL-15+mAb. Differences in the percentages at day 0 with respect to day 14 in IL-2 or IL-15 conditions, were evaluated using the Wilcoxon-Mann-Whitney test (*p<0.05; **p<0.01; ***p<0.001; ****p<0.0001) and statistical significances are reported. **(C, D)** Functional abilities of mAb-expanded NKG2C^+^CD57^+^ NK cells were evaluated at day 14 for both cytokine conditions (IL-2+mAb in **C** and IL-15+mAb in **D**). CD107a degranulation assays were performed against the indicated cell lines: Nalm-16, HT-29, Lovo, K562, LCL 721.221 wild type (221wt), transfected LCL 721.221 expressing HLA-E (221E), Raji and Rituximab-opsonized Raji (Raji+Rtx). Bars indicate the mean ± SD. Data were analyzed by Wilcoxon-Mann-Whitney test and statistical significances are indicated (**p<0.01; ***p<0.001; ****p<0.0001). **(E)** IFN-γ and TNF-α intracellular production capability of mAb-expanded NKG2C^+^CD57^+^ NK cells at day 14 were tested for both cytokine conditions (IL-2+mAb and IL-15+mAb) in co-cultures with the indicated cell lines: K562, LCL 721.221 wild type (221wt), transfected LCL 721.221 expressing HLA-E (221E), Raji and Rituximab-opsonized Raji (Raji+Rtx). Bars indicate the mean ± SD. Data were analyzed by Wilcoxon-Mann-Whitney test and statistical significances are reported (**p<0.01; ***p<0.001; ****p<0.0001). For clarity, statistical significances regarding the comparisons with the “NK alone” condition are indicated in the table on the right.

A variable fraction of adaptive NKG2C^+^ NK cells lacks the expression of the adaptor proteins FcεRγ and Syk due to epigenetic modifications induced by CMV infection (**Figure 4A** day 0) (22, 25, 26). Following mAb-mediated expansion, this peculiar characteristic was maintained, as indicated by the frequency of NKG2C^+^ CD57^+^ FcεRγ^–^ and Syk^–^ NK cells at day 14 (**Figure 4A** day 0 vs 14 and representative experiment in **Supplementary Figure S3A**). The expanded NKG2C^+^ CD57^+^ NK cells normally expressed the signaling molecule CD3ζ and the activating receptor CD16, although CD16 MFI was reduced in cultured cells as reported also in other expansion methods (34, 35, 37) (**Figure 4A**; **Supplementary Figure S3**), and were characterized by high levels of perforin and granzyme B, as expected for highly differentiated NK cells (not shown) (2). Importantly, the main activating NK receptors (i.e., NKp46, NKp30, NKp44, NKG2D, and DNAM-1) expression was maintained or upregulated compared to day 0 (**Figure 4B**; **Supplementary Figure S3B**). Notably, mAb-expanded adaptive NKG2C^+^CD57^+^ NK cells were highly functional in degranulation assays against tumor cell lines of different hystotypes, i.e. against K562, a classic NK cell susceptible HLA-I negative target, Lovo (HLA-I^-^), but also against HLA-I^+^ tumor cells as NALM-16 and HT-29 (**Figure 4C** IL-2+mAb and 4D IL-15+mAb).

As described previously, the lower FcεRγ expression in adaptive NK lymphocytes is associated with strong ADCC capabilities. Indeed, adaptive NKG2C^+^CD57^+^NKG2A^–^ self iKIR^+^ FcεRγ^–/low^ NK cells showed high degranulation levels in response to IgG-opsonized tumor cells (Rituximab-coated Raji), after expansion with mAb and IL-2 or IL-15 (**Figures 4C, D** and representative experiment in **Supplementary Figure S4**). Furthermore, such expanded NK cells were found to be highly functional also against a HLA-E^+^ transfected cell line (221E, **Supplementary Figure S5**; **Figures 4C, D**, **Supplementary Figure S4**). The high response against the HLA-E^+^ target indicates that the expression and function of the activating receptor CD94/NKG2C are well preserved during the CD94 mAb-driven expansion. The NKG2C^+^ CD57^–^ NK cell subset that moderately expanded in our experimental system (**Figure 2A**), displayed similar functional abilities (not shown). A modest degranulation was observed also when mAb+IL-15-induced adaptive NK cell expansions were tested against primary myeloid leukemic blasts (**Supplementary Figure S6**). Notably, degranulation levels were increased upon mAb-mediated masking of HLA-I molecules, by interrupting inhibitory KIR-HLA-I interactions between expanded adaptive NK cells and primary AML. This observation confirms the relevance of choosing a suitable donor mismatched with the HLA-I haplotype of the patient to achieve the highest killing potential of KIR^+^ adaptive NK cells.

Interestingly, mAb-expanded adaptive NKG2C^+^CD57^+^ NK cells were also capable of producing IFN-γ and TNF-α in response to K562, 221E and Rtx-Raji, with higher efficiency when NK cells were expanded in the presence of IL-15 (**Figure 4E**).

## 4 Discussion

The functional capabilities and phenotype diversity of human NK cell subpopulations have encouraged the design of novel immunotherapeutic approaches. In this context, the unique features of CMV-driven adaptive NK cells, i.e., strong ADCC response, highly differentiated profile, and longevity, make this NK cell subset highly suitable for cell-based immunotherapy. Notably, despite their virus-induced generation, adaptive NK cells demonstrated anti-tumor potential in transplanted leukemic patients where high adaptive NK cell numbers closely correlated with better survival (29–31). Indeed, clinical trials based on the infusion of adaptive NK lymphocytes are currently underway in patients suffering from acute myeloid leukemia (NCT03081780), and solid tumors, including ovarian and peritoneal carcinoma (NCT03213964), gastric, colorectal, breast, head and neck tumors (NCT03319459). However, the use of adaptive NK cells in the clinic requires further optimization of the methods to safely yield large numbers of cells with strong anti-tumor potential.

Here, we developed a novel method to expand adaptive CD94/NKG2C^+^ NK cells that combines the mAb-mediated engagement of CD94 to the stimulation provided by IL-2 or IL-15 cytokines and offers interesting advantages over previous protocols.

Similarly to other methods, our protocol requires the selection of CMV^+^ HD donors and a pre-NK cell enrichment from PB mononuclear cells; however, at variance with other approaches, a single T-cell depletion step is sufficient to selectively expand adaptive CD56^+^CD16^+^NKG2C^+^ NK cells, most of which co-express CD57. Importantly, expanded NKG2C^+^ NK cells lack the inhibitory receptor NKG2A, whose co-expression on expanded NK cells could decrease their cytotoxic responses and thus their immunotherapeutic potential. Indeed, although the frequency of NKG2C^+^ NKG2A^+^ PB-NK cells was low in PB-NK cells before culture (day 0, **Figure 3A**), the prolonged cytokine exposure might upregulate NKG2A, as occurred in a previously reported expansion method (36). Remarkably, our CD94-based expansion system favored the expansion of adaptive NKG2C^+^NKG2A^–^ NK cells thanks to the inhibitory signal delivered by the stimulation on NK cells expressing the inhibitory heterodimer CD94/NKG2A. A further advantage of our protocol is that expanded NKG2C^+^ NK cells do not upregulate PD-1, an immune checkpoint able to hamper the recognition and killing of PD-Ls^+^ tumor cells (17, 46). At variance with our method, the exposure to multiple proinflammatory cytokines (34, 36), as well as the prolonged mAb-mediated stimulation of NKG2C combined with IL-15, was shown to favor PD-1 upregulation (47), thus decreasing the NKG2C^+^ NK cell therapeutic potential.

The method described here also fulfills the need to expand adaptive NK cells with a given KIR specificity. Indeed, expanded adaptive NK cells expressed in most instances a single self iKIR specific for HLA-C1 (KIR2DL2-L3) or for HLA-C2 (KIR2DL1). The almost exclusive expression of a single inhibitory KIR in adaptive NK cells allows their exploitation as alloreactive effectors against HLA-I-positive tumors, such as acute leukemias. For this purpose, it will be necessary to select suitable CMV^+^ healthy donors bearing adaptive, educated NK cells characterized by an iKIR receptor recognizing self-HLA-I molecules absent in the patient. The absence of iKIR-HLA interactions will enhance the anti-leukemia effect of expanded adaptive NK cells (48, 49).

Adaptive NKG2C^+^ NK cells expanded by our method, besides maintaining their distinctive molecular traits, such as a low expression of FcεRγ, displayed high ADCC abilities in response to opsonized tumor targets, a requisite mandatory for mAb-mediated immunotherapies. Furthermore, the expanded cell population showed degranulation in the presence of HLA-E^+^ targets, indicating that the activating receptor CD94/NKG2C is still functional despite a prolonged stimulation that could induce cellular exhaustion (47). Moreover, mAb-expanded adaptive NK cells were active against both hematological and solid tumor cell lines and capable of producing IFN-γ and TNF-α at levels comparable to NK cells expanded according to previous methods (34). Future studies will investigate the metabolic requirements of CD94-expanded adaptive NK cells, also given the central role of metabolic fitness in NK cell function (50, 51).

A further advantage of our culture protocol is that, at variance with previous methods (34, 35, 37), it does not require feeder cells or genetically manipulated cell lines such as HLA-E^+^ cell transfectants. This makes our method safer and more suitable to be adapted to GMP standards and large-scale clinical applications. Indeed, the protocol described here represents a solid and reproducible method that can be further optimized to fully exploit its advantages, for example by the use of specialized culture media and/or bioreactors to obtain higher fold expansion rate of NKG2C^+^ NK cells and achieve cell numbers necessary for cell-based immunotherapies.

Expanded adaptive NK cells could be used for adoptive transfer in patients also in combination with mAbs or new biological tools to maximize their activity against tumors or to control viral infections (3, 4). Besides mAbs directed against tumor-associated antigens (e.g., CD20, EGFR, HER-2), innovative molecules, such as bi- or tri-specific antibodies or other NK cell engagers (NKCE), have been recently designed (33, 52–56). These molecules simultaneously bind a tumor-associated antigen and NK cell receptors including CD16 and NKp46 (6) enhancing and directing the cytotoxic activity against the tumor target. A further possibility could be to expand adaptive NK cells *in vivo* by administering mAb to patients to stimulate both the expansion of adaptive NK cells and their anti-tumor cytotoxic capabilities. The mAb could be engineered in its variable region (CDR), to generate more efficient and flexible tools for *in vivo* use, possibly containing a cytokine such as IL-15 supporting NK cell viability and proliferation. In addition, expanded adaptive NK cells could be engineered to generate long-living tumor-specific Chimeric Antigen Receptor-modified NK cell effectors (33, 57–59) to be used in cell-based precision medicine against hematological and solid tumors.

Our method to expand functional adaptive NK cells offers technical and economic advantages and can be used to achieve large-scale production of immune effectors cells for immunotherapeutic approaches.

## Data Availability

The original contributions presented in the study are included in the article or in the **Supplementary Material**. Additional raw data supporting the conclusions of this article will be made available by the authors, without undue reservation.
