# Supplementary material for: CD94-driven in vitro expansion of highly functional adaptive NKG2C+ NKG2A- CD57+ NK cells from CMV+ healthy donors
Source: Front Immunol. 2025 Jan 31;16:1481745. doi: 10.3389/fimmu.2025.1481745 (PMC11825780; doi:10.3389/fimmu.2025.1481745)
Supplement: Supplementary file 1 [file DataSheet1.pdf]

Supplementary Material

1 Supplementary Methods

Monoclonal antibodies, flow-cytometry analyses

The different monoclonal antibodies as well as the isotype-specific secondary reagents (goat anti-mouse) used in flow-cytometry analyses are shown in Supplementary Table 2 (Supplementary Table S2). When mAbs labeled with fluorochromes were used, appropriate isotype controls supplied by the same companies were added. In immunofluorescence experiments, cells were incubated with antibodies mixtures for 30 min at 4°C and washed with PBS supplemented with 5% FBS. NK cell phenotype and effector functions were analyzed selecting NK cells by physical parameters and by the combined use of anti-CD56, anti-CD16, anti-CD3, anti-CD19, anti-CD14 mAbs as depicted in the gating strategies in Fig. S1. To evaluate the different NKG2C/CD57/NKG2A/KIR subpopulations, the appropriate mixtures of mAbs reported in Table S1 were used. For intracellular flow-cytometry analyses, the cells were fixed and permeabilized with Foxp3 permeabilization kit (Miltenyi Biotec) according to the manufacturer's instructions. Before samples acquisition, the Fixable Viability Stain reagent (BD Biosciences) was added to each sample to exclude dead cells. Flow-cytometry analyses were performed on an eight-colors BD FACSVerse (Becton Dickinson, Mountain View, CA), and data were analyzed by FACSuite version 1.0.6 software.

KIR and KIR-ligand analyses

DNAs were extracted from selected PB samples using the QIAamp DNA Blood Mini kit (Qiagen, Hilden, Germany), and DNA concentrations, measured by the NanoDrop spectrophotometer (Thermo Fischer Scientific, Wilmington, DE, USA), were adjusted to 20ng/µL. *KIR* and *KIR-ligand* genes were analyzed using a sequence-specific primer-PCR (SSP-PCR) approach. In particular, the commercial kit KIR genotyping and KIR HLA Ligand (CareDx, Stockholm, Sweden) were used following the manufacturer's instructions. PCR reactions were performed using the Bio-Rad T100 Thermo Cycler, and the PCR products were resolved into a 1,5% agarose gel. Sanger sequencing was performed to discriminate between *KIR3DL1* alleles coding for expressed/functional receptors (i.e., carrying TCG codon 86) and those coding for polypeptides retained in the cytoplasm (TTG codon 86) as previously described (1). The presence of *KIR2DL3*004*, **005*, **010*, and **036* alleles, coding for receptors recognized by the 11PB6 mAb, was tested as previously reported (2).

AML patients and leukemic blasts isolation

PB samples from adult AML patients were provided by the Biological Resources Center-IRCCS Ospedale Policlinico San Martino (Genoa, Italy). The study was conducted in accordance with the local legislation and institutional requirements. All biological samples were collected after obtaining written informed consent from the patients, in adherence to the current regulations and the Declaration of Helsinki. Diagnosis was established by cytologic, molecular and immunophenotypic criteria. Patient's leukemic blasts named as AML1 was diagnosed a high-risk acute myeloid leukemia (HR AML), patient's leukemic blasts named as AML2 was diagnosed acute myelomonocytic leukemia post myelodysplastic syndrome (MDS), according to European leukemia Network 2022. Both AML1 and

AML2 leukemic blasts displayed a similar surface phenotype CD33+CD34+, CD14-, as reported in Supplementary Table S3.

Briefly, PB mononuclear cells (PBMCs) were separated by density gradient centrifugation (Ficoll-Hypaque solution, Sigma-Aldrich, St. Louis, MO) analyzed by immunofluorescence and flow-cytometry and frozen. Subsequently, PBMC were thawed and processed to obtain leukemic blasts-enriched preparations to be used as target cells in degranulation assays. Normal lymphocytes and monocytes were depleted from patients PBMC by anti-CD3, anti-CD19 and anti-CD14 magnetic microbeads (MACSmicrobeads Miltenyi Biotec, Bergisch Gladbach, Germany).

2 Supplementary Figures and Tables

2.1 Supplementary Figures

Supplementary Figure S1

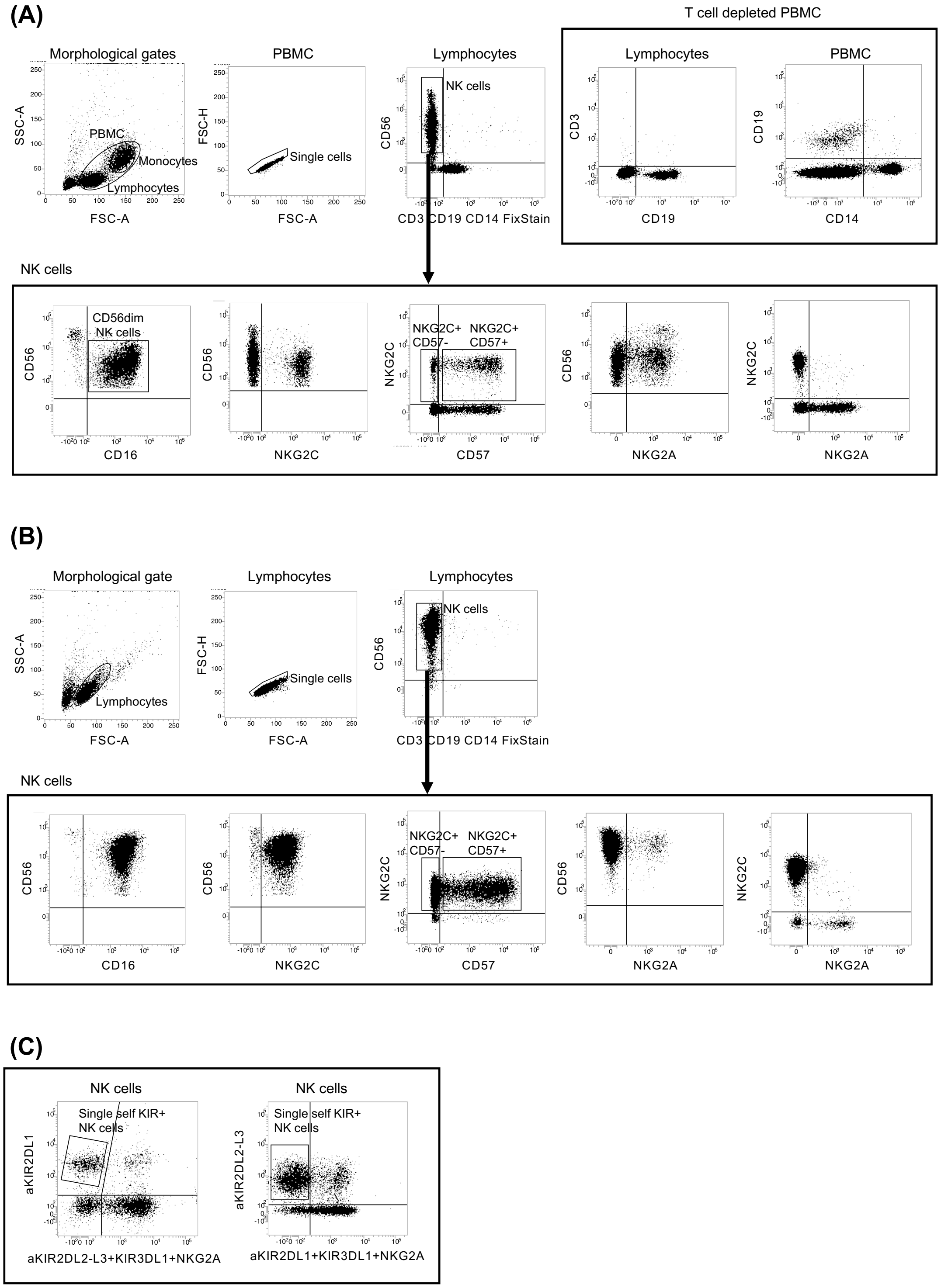

Supplementary Figure S1. Gating strategies for NK, T and B lymphocytes and monocytes

(**A**) PB-NK cells from healthy CMV$^+$ donors were identified, either before or after T cell-depletion, first by physical parameters, excluding any cell doublets. Subsequently, NK lymphocytes were selected as CD56$^+$ CD3$^-$ CD19$^-$ CD14$^-$ Fixable Viability Stain$^-$ (marker that identifies non-viable cells). CD3$^+$ T cells, CD19$^+$ B cells and CD14$^+$ monocytes were identified in T-depleted PBMC as shown in the corresponding box. NK cells were then analyzed for the expression of CD16 to identify CD56^{dim} NK cells, and of NKG2C combined to CD57 to identify adaptive NK cells, distinguishing NKG2C$^+$CD57$^-$ and NKG2C$^+$CD57$^+$ NK cell subpopulations for further analyses. NKG2A evaluation on NK cells is also indicated. (**B**) The gating strategy for activated NK lymphocytes is shown at day 10 of culture with the mAb and IL-2 or IL-15. NK cells were identified as CD56$^+$ CD3$^-$ CD19$^-$ CD14$^-$ Fixable Viability Stain$^-$ cells and essentially represented the only population found by the tenth day of culture. NKG2C$^+$CD57$^-$ and NKG2C$^+$CD57$^+$ adaptive NK cells were identified as in (A) and subsequently examined for the expression of specific markers and for their functional capabilities.
(**C**) The gating strategy for NK lymphocytes expressing a single self-iKIR and lacking NKG2A and other iKIR receptors is depicted for 2 representative donors characterized by a KIR A/A genotype (HD A, left and HD B, right, see Fig.S2). After selecting NK lymphocytes as in panels A and B, the frequency of NK lymphocytes expressing KIR2DL1 as the only HLA-I specific iKIR (recognizing HLA-C2) was evaluated in HD A by combining anti-KIR2DL1/S5-PE with a mixture of anti-KIR2DL2/L3/S2, anti-KIR3DL1 and anti-NKG2A antibodies in the same fluorescence channel (APC). The frequency of NK lymphocytes expressing KIR2DL2/L3 as the only HLA-I specific iKIR (recognizing HLA-C1) was measured in HD B by combining anti-KIR2DL2/L3/S2-PE with a mixture of anti-KIR2DL1/S5, anti-KIR3DL1 and anti-NKG2A antibodies in the same fluorescence channel (APC).

Supplementary Figure S2

	*KIR*s																	
HD	2DL1	2DL2	2DL3	2DL4	2DL5A	2DL5B	2DS1	2DS2	2DS3	2DS4	2DS5	3DL1	3DL2	3DL3	3DS1	2DP1	3DP1	KIR genotype
A												C/T						A/A
B												C/T						A/A
C												C/T						A/A
D												C						B/X
E																		B/X
F			*									C/T						A/A

KIR-L						
C1	C2	Bw4 T	Bw4 I	A Bw4	Bw6	self KIR expressed on NKG2C+CD57+ NK cells
						KIR2DL1
						KIR2DL3
						KIR2DL3
						KIR2DL2/L3
						KIR2DL2/L3
						KIR2DL3

Supplementary Figure S2. Analysis of *KIR* and *KIR-ligand* genes.

The presence (grey boxes) or absence (white boxes) of the indicated *KIR* and *KIR-ligand* genes are reported. In *KIR2DL3* boxes, * indicates the presence of at least one of the following *KIR2DL3* alleles: **004*, **005*, **010*, and **036*. The presence of *KIR3DL1* alleles characterized by TCT (C) or TTG (T) codon 86, coding for surface receptors or polypeptide retained into the cell, respectively, are indicated. Based on the results, *KIR* genes coding for iKIRs recognizing self HLA-I have been identified (dark

grey). For each donor the self KIR expressed by adaptive NKG2C$^+$CD57$^+$ cells before and after expansion is indicated.

Supplementary Figure S3

A

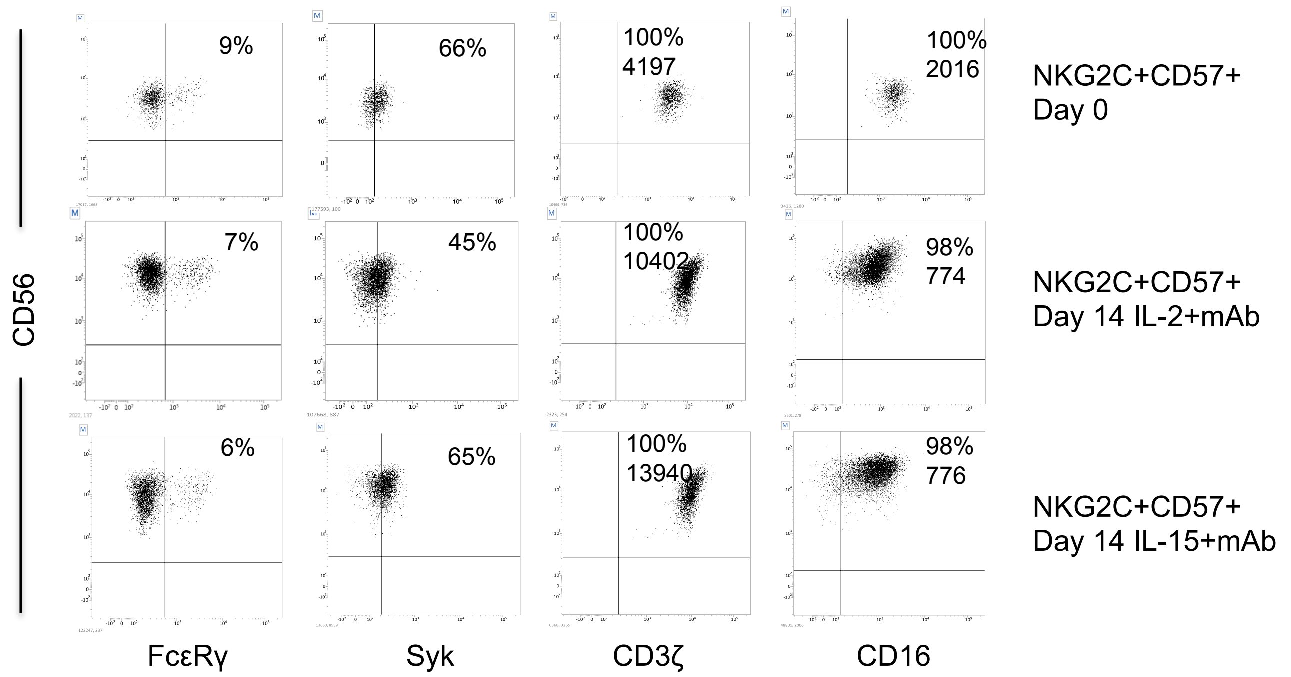

B

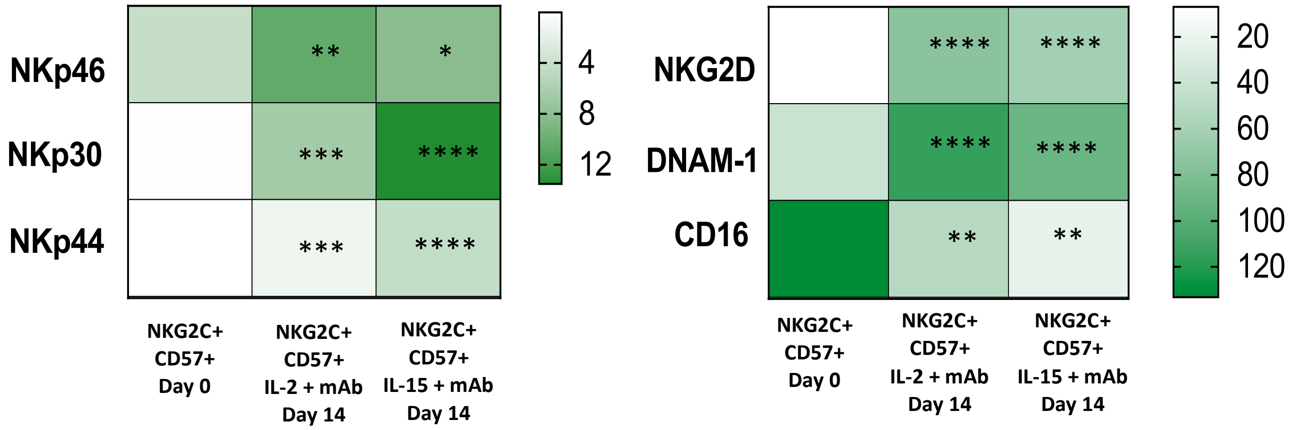

Supplementary Figure S3. MAb-expanded adaptive NKG2C$^+$CD57$^+$ NK cells maintain their intracellular molecular signature and show increased levels of NCRs, NKG2D and DNAM-1

(**A**)The expression of the intracellular signaling molecules FcεRγ, Syk, and CD3ζ together with surface CD16 expression in NKG2C$^+$CD57$^+$ NK cells are shown for a representative donor at day 0 compared

to day 14 in IL2+mAb or IL-15+mAb. Percentages of positive cells and/or MFI values are indicated in each plot.

(B) Heatmap depicting the normalized Median Fluorescence Intensity of the indicated activating receptors (NKp46, NKp30, NKp44 left panel and NKG2D, DNAM-1 right panel) in NKG2C^{+}CD57^{+} NK cells at day 0 and 14 in IL2+mAb or IL-15+mAb. Differences in nMFI at day 0 with respect to day 14 in IL-2 or IL-15 conditions, were evaluated using the Wilcoxon-Mann-Whitney test (*p<0.05; **p<0.01; *** p<0.001; **** p<0.0001) and statistical significances are reported.

Supplementary Figure S4

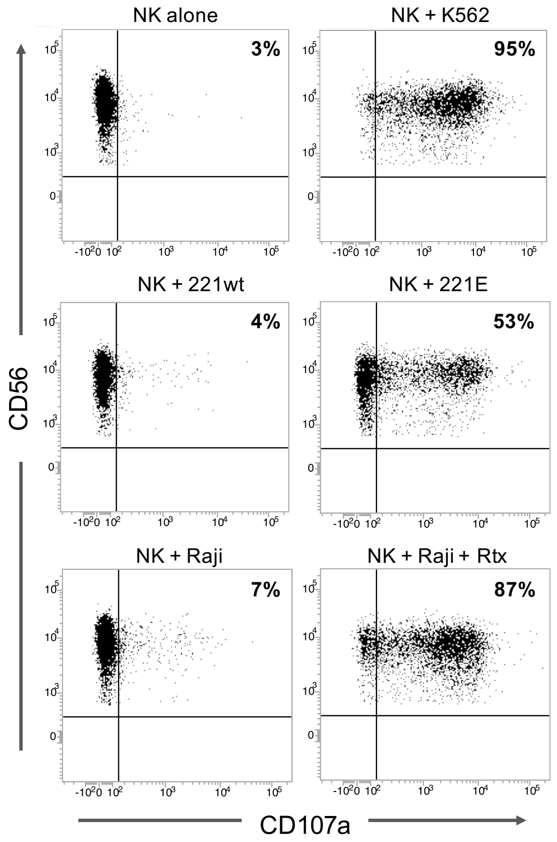

Supplementary Figure S4. MAb-expanded NKG2C^{+}CD57^{+} adaptive NK cells show high functional capabilities.

A representative degranulation experiment with NK cells expanded in IL-2+mAb is shown, in which percentage of surface CD107a expression on NKG2C^{+}CD57^{+} NK cells is indicated in the top right quadrant for each condition.

Supplementary Figure S5

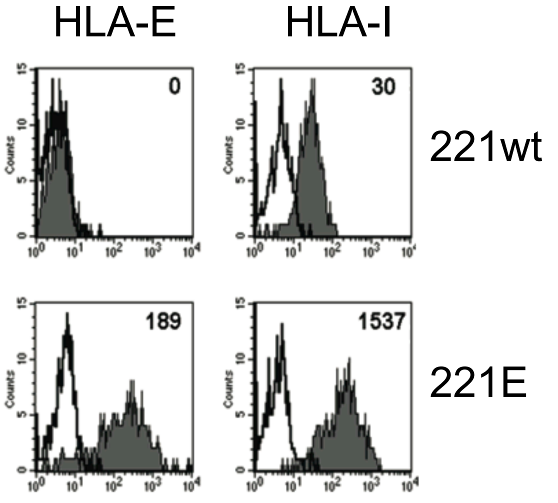

Supplementary Figure S5. HLA-E expression on transfected cell line

221wt and 221E were analysed by flow cytometry for the expression of both non-classic HLA-E molecules (3D12 mAb) and HLA-class I (HLA-I) molecules (A6/136 mAb). Empty histograms represent cells incubated with the secondary reagent only. Mean Fluorescence Intensity values are indicated in each histogram plot.

Supplementary Figure S6

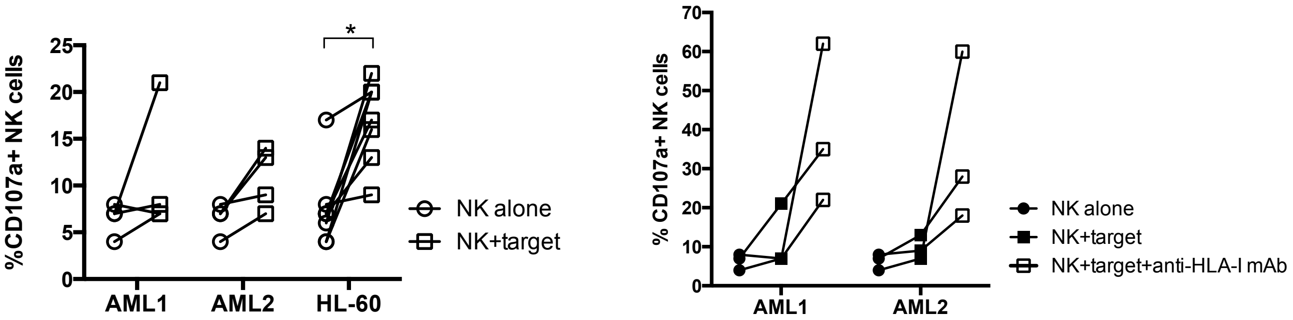

Supplementary Figure S6. Degranulation capabilities of mAb-expanded NKG2C$^+$CD57$^+$ NK cells against primary acute myeloid leukemia cells.

Functional abilities of IL15+mAb-expanded NKG2C$^+$CD57$^+$ NK cells were evaluated at day 14 against two different primary samples of acute myeloid leukemia (AML1 and AML2) (n=4) in parallel to HL-60 AML cell line (n=8) (left). On the right, n=3 degranulation experiments against AML1 and AML2 were performed in the presence of anti-HLA-I mAb (empty squares). Data were analyzed using the Wilcoxon-Mann-Whitney paired test and statistical significances are reported (*p<0.05).

2.2 Supplementary Tables

Supplementary Table S1. Frequencies of NKG2C/CD57 NK cell subsets

Selected CMV+ HD	%NKG2C+ CD56^{dim} NK	%NKG2C+CD57- CD56^{dim} NK	%NKG2C+CD57+ CD56^{dim} NK
A	29	10	19
B	67	5	62
C	35	8	27
D	42	5	37
E	25	6	19
F	40	2	38
G	48	7	41
H	30	7	23
I	20	3	17
L	40	10	30

List of abbreviations: CMV+= cytomegalovirus positive serostatus; HD= healthy donors

Supplementary Table S2. List of flow cytometry antibodies

Antigen	Fluorochrome	Clone	Source
**CD3**	VioGreen	REA613	Miltenyi Biotec
**CD3**	PerCP-Cy5.5	UCHT1	BD Biosciences
**CD3ζ**	PE	6B10.2	eBioscience Inc.
**CD14**	VioGreen/APC	REA599	Miltenyi Biotec
**CD16**	PerCP-Cy5.5	3G8	BD Biosciences
**CD19**	VioGreen/VioBlue	REA675	Miltenyi Biotec
**CD33**	VioGreen	REA775	Miltenyi Biotec
**CD34**	APC	QBEnd10	R&D Systems
**CD34**	PE	QBEnd10	Beckman Coulter
**CD48**	-	CO202	Produced in our laboratory
**CD56**	PC7	N901	Beckman Coulter
**CD56**	-	C218	Produced in our laboratory
**CD57**	VioBlue	TB03	Miltenyi Biotec
**CD107α (LAMP1)**	PE/APC-H7	H4A3	BD Biosciences
**FcεRI-γ**	FITC	Polyclonal	Merck Millipore
**Fixable Viability Stain**	BV510	-	BD Biosciences
**Granzyme B**	PE	GB12	Invitrogen
**Goat anti-mouse IgG1**	PE	Polyclonal	SouthernBiotech
**Goat anti-mouse IgG2b**	PE/APC-Cy7	Polyclonal	SouthernBiotech
**HLA-E**	-	3D12	Abnova
**HLA-DR**	-	D1.12	Produced in our laboratory
**HLA-G**	-	MEM-G/9	Abnova
**HLA-I**	-	W6/32	Produced in our laboratory
**IFN-γ**	APC/PE	B27	BD Biosciences
**KIR2DL1/S1-2DL3*005**	FITC/APC	11PB6	Miltenyi Biotec
**KIR2DL1/S5**	FITC/APC/PE	143211	R&D Systems

**KIR2DL2/L3/S2**	APC	DX27	Miltenyi Biotec
**KIR2DL2/L3/S2**	FITC	CH-L	BD Biosciences
**KIR2DL2/L3/S2**	PE	GL183	Beckman Coulter
**KIR3DL1**	FITC	DX9	Miltenyi Biotec
**LAG-3**	APC	REA351	Miltenyi Biotec
**LFA-3**	-	TS2/9	Produced in our laboratory
**MIC-A**	-	BAM195	Kindly provided by Prof. Pende
**Nectin2**	-	L14	Produced in our laboratory
**NKG2A**	APC	Z199	Beckman Coulter
**NKG2C**	-	134522	R&D Systems
**NKG2C**	APC	134591	R&D Systems
**PD-1**	PE	PD1.3.1.3	Miltenyi Biotec
**Perforin**	PerCP-Cy5.5	DG9	BD Biosciences
**PVR**	-	5A.10	Produced in our laboratory
**Syk**	FITC	4D10	BD Biosciences
**TIGIT**	-	741182	R&D Systems
**TIM-3**	APC	REA635	Miltenyi Biotec
**TNF-α**	PE	REA656	Miltenyi Biotec
**ULBP1**	-	170818	R&D Systems
**ULBP2**	-	165903	R&D Systems
**ULBP3**	-	166510	R&D Systems
**ULBP4**	-	709116	R&D Systems

Supplementary Table S3.

Marker	AML1	AML2
CD33	+	+
CD34	+	+
CD14	-	-
HLA-I	+	+
HLA-E	-	-
HLA-G	-	-
HLA-DR	+	+
LFA-3	+	+
PVR	+	+
Nectin-2	+	+
MIC-A	-	-
ULBP1	-	-
ULBP2	-	-
ULBP3	-	-
ULBP4	-	-
CD48	-	-
CD56	+	-
NKG2C	-	-

3 Supplementary References

1) Alicata C, Pende D, Meazza R, Canevali P, Loiacono F, Bertaina A et al. Hematopoietic stem cell transplantation: Improving alloreactive Bw4 donor selection by genotyping codon 86 of KIR3DL1/S1. Eur J Immunol. 2016;46(6):1511-7.

2) Meazza R, Falco M, Canevali P, Loiacono F, Colomar-Carando N, Muntasell A, Rea A, et al. Characterization of KIR+ NK cell subsets with a monoclonal antibody selectively recognizing KIR2DL1 and blocking the specific interaction with HLA-C. HLA. 2022;100(2):119-132.
